# Notes and News

**Published:** 1894-03-17

**Authors:** 


### Notes and News.

Collected and Communicated.

### MEETINGS OF THE WEEK.

Royal Hospital for Diseases of the Chest, City Road: Annual General Meeting, March 14th, 3 p.m.

Earlswood Asylum: Festival Dinner, Hôtel Métropole, March 15th.

Royal Humane Society, 4, Trafalgar Square: Annual Meeting, March 15th, 4.30 p.m.

Dental Hospital, Leicester Square: Annual meeting, March 15th, 5.30 p.m.

St. Marylebone General Dispensary: Re-opening, March 16th, 3 p.m.

St. Mary's Hospital, Paddington: Annual Meeting, March 17th, 4.30 p.m.

Home for Incurable Children, Maida Vale: Annual Meeting, March 17th, 3 p.m.

Chelsea Hospital for Women: Special Meeting, March 19th, 3 p.m.

---

We are glad to learn that the Hospital Sunday Fund has received a most substantial legacy. Mr. Ingham Whittaker has bequeathed to it £5,000.

---

The Joint Hospital Board for Acton, Chiswick, and Hanwell have applied for a provisional order to acquire a site for an infectious hospital at Perwale, near Harrow. The choice of this site is provoking the usual opposition in the neighbourhood in which it is situated.

---

The foundation-stone for a new infectious hospital at Birkenhead has been laid by the Mayor of that town. The hospital is to accommodate 44 patients. It is to be built on the pavilion plan. The grounds consist of five and a half acres, and provision is made for extension of the buildings if required.

---

The *City of Adelaide*, purchased by the Southampton Borough Authorities for conversion into an isolation hospital, is now ready for occupation. The cost of furnishing has been £5,500. The ship has been open for inspection during several days, and the Health Committee have been holding receptions on board to celebrate the event.

---

Dr. Gilbart Smith has been appointed full physician to the London Hospital vice Dr. Hughlings Jackson, who has become consulting physician to that institution. There are few men more popular than Dr. Gilbart Smith, a fact which was eloquently apparent from the unanimity which characterised the proceedings at the quarterly Court of Governors at which these appointments were made.

---

An alarming outbreak of small-pox has occurred in the Essex Lunatic Asylum. Eight cases have proved fatal out of the 38 cases reported. It would seem that no proper means of isolation exist in connection with the asylum, or so large a number are not likely to have fallen victims to the epidemic. The outbreak has been confined to the male wards, patients and attendants alike being attacked.

---

The National Hospital for Consumption in Ireland is so far advanced that the board of governors have signed a contract for the erection of the institution near Newcastle, in County Wicklow, for £8,956. The plan provides a building for 100 patients, and an administrative block. It is intended to commence with the administrative block, and two residential blocks for twelve patients in each.

---

Active measures are to be taken at Newcastle to improve the financial condition of the Royal Infirmary. In order to effect this an industrial exhibition is to be arranged for the coming autumn under the management of experienced organisers. The management is everything in a case like this, and if successful, the exhibition will prove of substantial help to the infirmay and benefit the neighbourhood as well.

---

St. Bartholomew's Hospital is to extend its benefits not only to the sick, but to the well amongst the population also. The authorities own the enclosure known as St. Bartholomew's Square, and this they have handed over to the Metropolitan Public Gardens Association for eight years, at a nominal rental of one shilling per annum, to be paid by the local vestry, who will undertake the charge after the Gardens Association have laid out the space as a playground for the children of the neighbourhood.

---

At the end of February Ilkeston became possessed of a new hospital, the opening ceremony, at which Lord Belper presided, being made a demonstration of the interest felt in the neighbourhood for the hospital. The old hospital, erected in 1884 with ten beds, proved quite inadequate to the needs of the neighbourhood. The new hospital is built on an excellent site, presented by Mr. E. M. Mundy, J.P. It contains 26 beds, dispersed over male, female, and children's wards. The buildings have been erected at a cost of £3,000, and are handsome and well fitted. The institution will be opened free of debt, all the funds except those for furnishing having been already contributed. The appeal for necessary funds was responded to in a hearty manner, the working classes and miners being especially generous in their contributions.

